# Cyanobacterial symbionts diverged in the late Cretaceous towards lineage-specific nitrogen fixation factories in single-celled phytoplankton

**DOI:** 10.1038/ncomms11071

**Published:** 2016-03-22

**Authors:** Francisco M. Cornejo-Castillo, Ana M. Cabello, Guillem Salazar, Patricia Sánchez-Baracaldo, Gipsi Lima-Mendez, Pascal Hingamp, Adriana Alberti, Shinichi Sunagawa, Peer Bork, Colomban de Vargas, Jeroen Raes, Chris Bowler, Patrick Wincker, Jonathan P. Zehr, Josep M. Gasol, Ramon Massana, Silvia G. Acinas

**Affiliations:** 1 Department of Marine Biology and Oceanography, Institut de Ciències del Mar (ICM), CSIC, Pg. Marítim de la Barceloneta 37-49, 08003 Barcelona, Spain; 2 School of Geographical Sciences, University of Bristol, Bristol BS8 1SS, UK; 3 Department of Microbiology and Immunology, Rega Institute KU Leuven, Herestraat 49, 3000 Leuven, Belgium; 4 VIB Center for the Biology of Disease, VIB, Herestraat 49, 3000 Leuven, Belgium; 5 Department of Applied Biological Sciences (DBIT), Vrije Universiteit Brussel, Pleinlaan 2, 1050 Brussels, Belgium; 6 Aix Marseille Université CNRS IGS UMR 7256, 13288 Marseille, France; 7 CEA-Institut de Génomique, Genoscope, Centre National de Séquençage, 2 rue Gaston Crémieux, CP5706, F-91057 Evry, France; 8 European Molecular Biology Laboratory, Structural and Computational Biology Unit, Meyerhofstrasse 1, 69117 Heidelberg, Germany; 9 Max-Delbrück-Centre for Molecular Medicine, 13092 Berlin, Germany; 10 CNRS, UMR 7144, Station Biologique de Roscoff, Place Georges Teissier, 29680 Roscoff, France; 11 Sorbonne Universités, UPMC Université Paris 06, UMR 7144, Station Biologique de Roscoff, Place Georges Teissier, 29680 Roscoff, France; 12 Ecole Normale Supérieure, PSL Research University, Institut de Biologie de l'Ecole Normale Supérieure (IBENS), CNRS UMR 8197, INSERM U1024, 46 rue d'Ulm, F-75005 Paris, France; 13 Université d'Evry, UMR 8030, CP5706 Evry, France; 14 Centre National de la Recherche Scientifique (CNRS), UMR 8030, CP5706 Evry, France; 15 Department of Ocean Sciences, University of California, Santa Cruz, California 95064, USA

## Abstract

The unicellular cyanobacterium UCYN-A, one of the major contributors to nitrogen fixation in the open ocean, lives in symbiosis with single-celled phytoplankton. UCYN-A includes several closely related lineages whose partner fidelity, genome-wide expression and time of evolutionary divergence remain to be resolved. Here we detect and distinguish UCYN-A1 and UCYN-A2 lineages in symbiosis with two distinct prymnesiophyte partners in the South Atlantic Ocean. Both symbiotic systems are lineage specific and differ in the number of UCYN-A cells involved. Our analyses infer a streamlined genome expression towards nitrogen fixation in both UCYN-A lineages. Comparative genomics reveal a strong purifying selection in UCYN-A1 and UCYN-A2 with a diversification process ∼91 Myr ago, in the late Cretaceous, after the low-nutrient regime period occurred during the Jurassic. These findings suggest that UCYN-A diversified in a co-evolutionary process, wherein their prymnesiophyte partners acted as a barrier driving an allopatric speciation of extant UCYN-A lineages.

## Abstract (web summary)

Nitrogen fixation in oceans is facilitated by associations between marine phytoplankton and cyanobacteria such as UCYN-A. Here, Cornejo-Castillo *et al*. show that UCYN-A diversified in the late Cretaceous under strong purifying selection to become lineage-specific symbiont partners with different prymnesiophytes.

## Graphical abstract

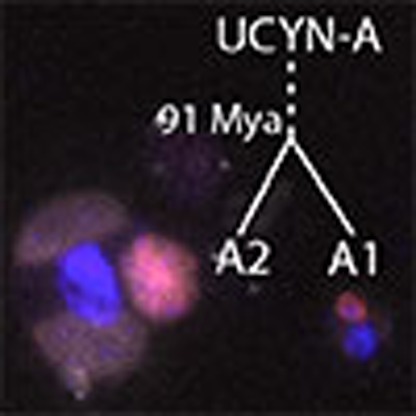

Symbiotic relationships involving diazotrophic microorganisms, that is, those capable of converting dissolved dinitrogen gas into ammonia, are of relevant interest in marine biogeochemistry because they represent major sources of fixed nitrogen, a limiting nutrient for primary production in the world's oceans^1^. As such, identifying these interactions is essential for understanding the role of symbiosis in biogeochemical cycles. Fortunately, the application of novel approaches such as high-throughput sequencing and single-cell genomics has greatly accelerated the pace of microbial symbiosis research^2^,^3^. This is notable in the case of *Candidatus Atelocyanobacterium thalassa* (UCYN-A), a unicellular diazotrophic cyanobacterium, and its partner, a single-celled eukaryotic alga of the class Prymnesiophyceae^4^. Prymnesiophytes as well as UCYN-A are abundant and widely distributed members of the marine plankton and represent ecologically relevant players in carbon and nitrogen cycles^5^,^6^,^7^,^8^,^9^. The streamlined genome of UCYN-A and the striking lack of genes encoding the photosystem II complex, the Calvin/Benson/Bassham cycle for carbon fixation, as well as other essential pathways such as the tricarboxylic acid cycle, hinted at a symbiotic lifestyle^10^,^11^,^12^. UCYN-A is now known to establish a mutualistic relationship based on the exchange of fixed carbon and nitrogen with two different cell-sized prymnesiophyte partners, the unicellular alga *Braarudosphaera bigelowii* (7–10 μm)^13^,^14^ and an uncultured closely related prymnesiophyte (1–3 μm)^4^,^15^.

Phylogenomic analyses have demonstrated the monophyly of UCYN-A within the marine cyanobacteria clade that includes *Crocosphaera* sp. and *Cyanothece* sp. clades^12^. Phylogenetic analysis of the UCYN-A nitrogenase gene (*nifH*) sequences, a common marker used to address the diversity of N_2_-fixing microorganisms, distinguished at least three distinct UCYN-A clades: UCYN-A1, UCYN-A2 and UCYN-A3 (ref. 14). Comparative genomics revealed that UCYN-A1 and UCYN-A2 lineages share largely syntenic genomic structures, suggesting that both lineages diverged after genome reduction from a common ancestor^12^. Yet, their time of evolutionary divergence and evolutionary pressures remain unknown. It has been suggested that these two variants could be adapted to different niches, that is, coastal waters (*B. bigelowii*) and open ocean (its closely related prymnesiophyte)^14^, but this ecological differentiation was recently ruled out^9^. Although the two prymnesiophyte partners could follow different ecological strategies^9^, the partner fidelity has never been tested in this symbiotic system, and therefore we cannot assume a similar ecological niche for their symbionts. Comparative gene expression studies could help to disentangle the ecological distinction of these two UCYN-A lineages but they are scarce and solely focused on the *nifH* gene expression without showing a clear differentiation in lineage-specific patterns^14^.

By designing and applying new probes in double catalysed reporter deposition fluorescence *in situ* hybridization (CARD-FISH), we identified the specific symbiotic associations at the UCYN-A lineage level in samples from South Atlantic waters from the *Tara* Oceans expedition, where we had previously verified significant abundances of the prymnesiophyte partners. The new probes allowed us to differentiate both symbiotic systems that resulted to vary in the number of UCYN-A cells involved. The coupled analyses of metagenomes and metatranscriptomes from surface and deep chlorophyll maximum (DCM) depths that encompassed four different plankton size fractions distinguish different prymnesiophyte partners based on difference in cell sizes captured in different size fractions, complementing and extending the results obtained by CARD-FISH. Gene expression was explored in the two UCYN-A lineages to decipher whether distinct lineages, in association with distinct partners, exhibit different expression patterns. Finally, we investigated the evolutionary pressures acting on UCYN-A1 and UCYN-A2 lineages by comparative genomic analyses and performed phylogenomic analyses to estimate the age divergence of the two symbiotic lineages. Our findings support a symbiont–host co-evolutionary scenario in the marine environment originating from a single ancestral symbiotic event in the late Cretaceous from which at least two different UCYN-A lineages diversified to become lineage-specific nitrogen fixation factories in their prymnesiophyte partners. Together, these investigations improve our understanding of the relevance of co-evolutionary processes in marine ecosystems and the ecological significance of N_2_-fixing symbiosis in the marine biogeochemical cycles.

## Results and Discussion

### Partner fidelity of the two UCYN-A lineages

**Figure 1:**
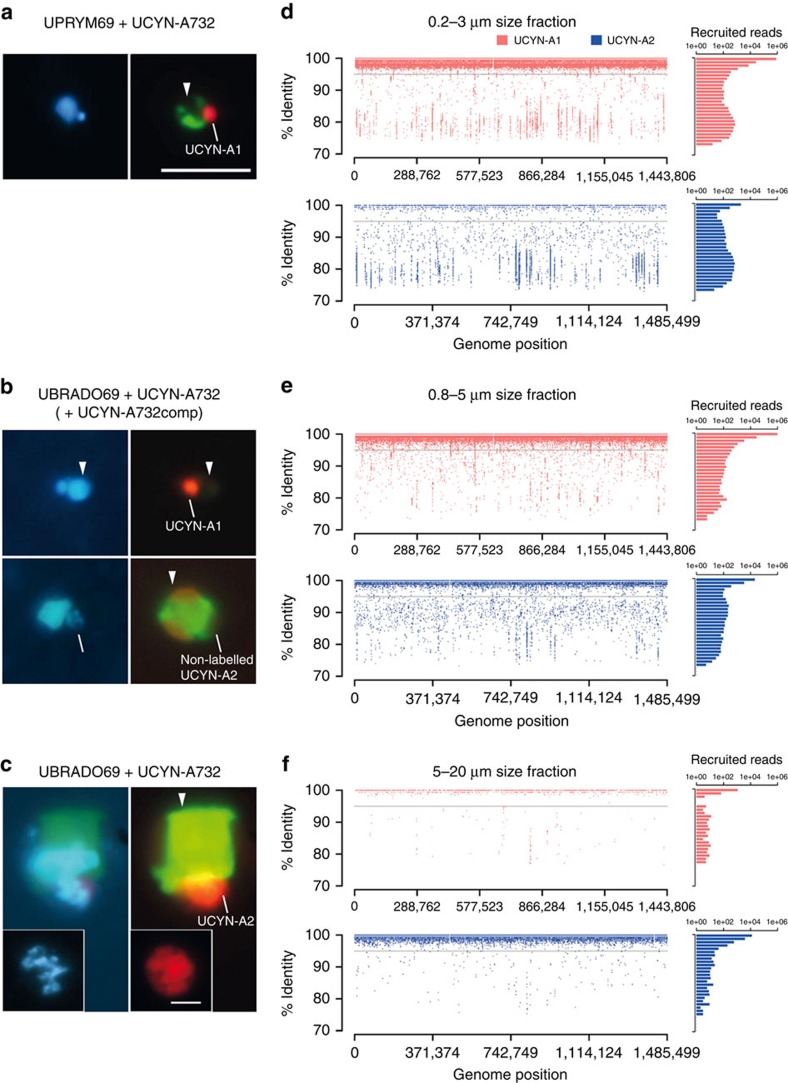
Partner specificity and variation of UCYN-A lineages with plankton size fraction. (**a**–**c**) Epifluorescence microscopy images with the double-CARD-FISH assay showing the specificity of symbiont–host pairs and (**d**–**f**) fragment recruitment of UCYN-A lineages in size-fractionated metagenomes from surface waters collected in station TARA_078. (**a**–**c**) Left panels correspond to the 4′,6-diamidino-2-phenylindole signal (blue-labelled DNA); right panels correspond to the combined signal of the prymnesiophyte-specific probes (green-labelled host under blue light excitation) and the UCYN-A probe (red-labelled symbiont under green-light excitation). (**a**) UCYN-A1 with its prymnesiophyte partner; (**b**) the two UCYN-A symbiotic pairs, indicating the specific labelling of UCYN-A1 (upper) and *B. bigelowii* (lower) with their specific partners, the small prymnesiophyte closely related to *B. bigelowii* and UCYN-A2 respectively; (**c**) *B. bigelowii* with UCYN-A2. The inset in **c** shows the detail of non-associated UCYN-A2 cells within a common symbiotic structure. Prymnesiophyte partners are indicated by arrow heads. Scale bar in **a** represents 5 μm and this scale is shared in **a**–**c** except in the inset of **c** where it indicates 2 μm. (**d**–**f**) On the left side, recruitment of metagenomic reads using UCYN-A1 and UCYN-A2 genomes as reference. Reads are plotted as red (UCYN-A1) or blue (UCYN-A2) dots depending on the closest hit genome, representing the covered genome positions (*x* axis) and the % of identity with the closest reference (*y* axis). A horizontal grey line set at 95% indicates the threshold for reads representing members of the same population as the reference genome. On the right side, histograms represent the number of recruited reads, in logarithmic scale, by UCYN-A1 (red) or UCYN-A2 (blue) genomes in intervals of 1% identity, from 100 to 70% identity.

Microscopic *in situ* identification of different UCYN-A lineages as well as their prymnesiophyte partners by specific CARD-FISH staining is crucial to determine the specificity of their relationships. The CARD-FISH method has been successfully applied to identify unicellular diazotrophic cyanobacteria^16^ as well as specifically targeting the UCYN-A clade^15^,^17^. However, to our knowledge there was not any reported probe to distinguish UCYN-A at the lineage level. We designed a competitor probe to be used with the UCYN-A732 probe^15^ to distinguish UCYN-A1 and UCYN-A2 lineages (Fig. 1a–c; Supplementary Table 1). Similarly, we designed two probes to distinguish the two prymnesiophyte partners, *B. bigelowii* (UBRADO69 probe) and the closely related prymnesiophyte (UPRYM69 probe) (Fig. 1a–c; Supplementary Table 1). The UCYN-A732 probe, in the absence of its competitor, labelled UCYN-A cells inside either *B. bigelowii* or the closely related prymnesiophyte partner (Fig. 1a,c). However, when the UBRADO69 probe was applied with the UCYN-A732 probe together with its competitor, UCYN-A cells were unlabelled or labelled when accompanying *B. bigelowii* or the closely related prymnesiophyte partner, respectively (Fig. 1b). It has been proposed that smaller UCYN-A cells are associated with smaller prymnesiophyte cells and vice versa, indicating different growth stages^17^. However, those findings were interpreted from microscopic observations of the UCYN-A symbiosis detected with the general prymnesiophyte PRYM02 and UCYN-A732 (without its competitor) probes, that is, without the ability to distinguish UCYN-A1 and UCYN-A2 cells. The results presented here show that both prymnesiophyte partners are phylogenetically closely related but distinct species, and therefore we suggest that the observed differences in cell sizes of prymnesiophyte partners reflect distinct species rather than different growth stages of the same species. These results demonstrate that UCYN-A lineages display partner fidelity with their prymnesiophyte partners, being *B. bigelowii* and the closely related prymnesiophyte in specific association with UCYN-A2 and UCYN-A1 lineages, respectively.

### The number of UCYN-A cells per partner is lineage specific

Previous studies have shown that the prymnesiophyte partners can harbour one or two UCYN-A cells^4^,^9^,^13^,^15^, pointing to a coupling between the prymnesiophyte cell division and the number of symbiotic cells, at least for UCYN-A1 (ref. 9). In our samples, only one UCYN-A1 cell per prymnesiophyte cell was detected (Fig. 1a,b). By contrast, *B. bigelowii* carried a symbiosome-like compartment with a variable but higher number of UCYN-A2 cells (∼3–10 cells) (Fig. 1b,c). This structure was observed both attached to the host and in a free state, as an entity composed by several UCYN-A2 cells enclosed by a common envelope (Fig. 1c). In a previous study, the UCYN-A2 cells found in *B. bigelowii* were separated from the *B. bigelowii* cytoplasm by a single membrane, likely a perisymbiont membrane, and the envelope of the UCYN-A2 itself consisted of three layers, possibly an outer membrane, a peptidoglycan wall and a plasma membrane^13^. Although UCYN-A1 and UCYN-A2 are very similar in terms of gene content, the genes involved in cell wall biogenesis and cell shape determination appear to be only present in UCYN-A2, suggesting clear structural differences associated with its host^12^. Therefore, our observations hint at different symbiotic organizations: while the UCYN-A1 lineage has one or two separated cells per host, the UCYN-A2 lineage may harbour up to 10 cells per prymnesiophyte partner cell within a common symbiotic structure.

### UCYN-A lineages vary in different plankton size fractions

A total of eight marine metagenomes from stations TARA_078 and TARA_076 were analysed to assess the distribution of UCYN-A lineages in several plankton size fractions (0.2–3, 0.8–5, 5–20 and >0.8 μm) of the microbial assemblages in surface and DCM waters (Table 1). We used the two UCYN-A genomes sequenced to date as reference genomes^11^,^12^ in the fragment recruitment of these metagenomic samples (Table 1). Because of the UCYN-A partner fidelity displayed by double CARD-FISH (see above), metagenomic sequence reads from UCYN-A lineages should vary with size fraction as predicted by the different cell sizes of the prymnesiophyte partners. The sequence reads from the UCYN-A1 lineage were primarily present in surface waters within the size fraction range of the small prymnesiophyte partner (0.2–3, 0.8–5 and >0.8 μm; Table 1). Almost 100% of the UCYN-A1 genome was recovered in each of the metagenomes from surface of these size fractions in the two stations. Likewise, UCYN-A1 sequence reads were poorly represented in the 5–20 μm size fraction (∼10% of genome recovery; Fig. 1d–f; Table 1). On the other hand, in TARA_078, the UCYN-A2 sequence read distribution in surface waters was consistent with the *B. bigelowii* cell size, that is, UCYN-A2 reads were nearly absent in the 0.2–3 μm size fraction metagenomes, but were more abundant in the 0.8–5, 5–20 and >0.8 μm fractions. In all these larger fractions, the UCYN-A2 reached high genome recovery values (90%, 76% and 99%, respectively), except for the >0.8 μm fraction in TARA_076 where UCYN-A2 was virtually absent (Fig. 1d–f, Table 1). In the >0.8 μm size fraction, UCYN-A1 was approximately nine times more abundant than UCYN-A2 in TARA_078 (Table 1). Likewise, in the same station, the small prymnesiophyte partner was more abundant than *B. bigelowii* based on V9 18S ribosomal RNA (rRNA) tags^9^. In the DCM samples, both UCYN-A lineages were poorly represented in the metagenome sequences, accounting for <14% and 1% of genome recovery for UCYN-A1 and UCYN-A2, respectively (Table 1). The same vertical distribution has been observed for their prymnesiophyte partners that were found preferentially in surface layers, while the rest of the prymnesiophyte assemblage peaked at the DCM^9^. Therefore, although the UCYN-A1 lineage was in general more abundant than UCYN-A2, a transition from the UCYN-A1 to UCYN-A2 lineage was observed from smaller to larger size fractions, likely explained by the partner fidelity and the difference in cell size of their prymnesiophyte partners.

Another interesting finding was that most of the metagenomic (and metatranscriptomic) reads mapping to the UCYN-A1 or UCYN-A2 genomes had very high sequence identities (>99% to their respective reference genome; Fig. 1d–f), which suggests an extremely low microdiversity within populations that were sampled from geographically distant regions in the Pacific (ALOHA and SIO) and South Atlantic Oceans (this study). The size-fractionated sampling strategy combined with the metagenomic analyses reported in this study will be also important to uncover the genomic pool of new UCYN-A lineages, such as UCYN-A3, to identify the lineage-specific distribution of UCYN-A populations and to set the cell size range of their partners, a first step for their identification.

### UCYN-A expression is streamlined to fuel nitrogen fixation

**Figure 2:**
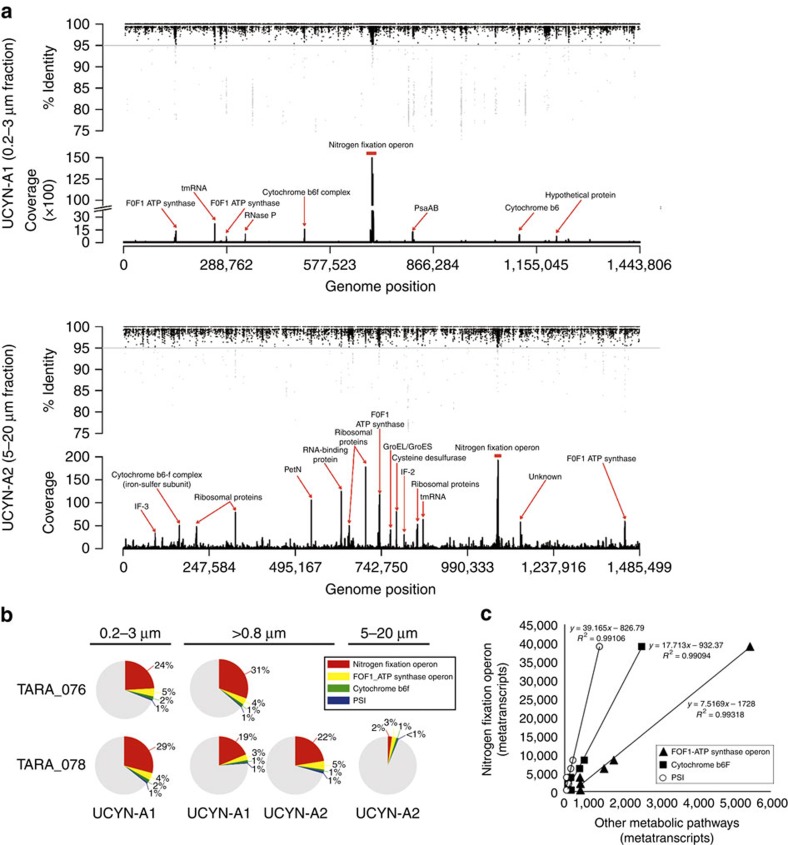
Genome expression in UCYN-A1 and UCYN-A2 lineages. (**a**) Metatranscriptome recruitment at the surface of the TARA_078 station of UCYN-A1 (0.2–3 μm) and UCYN-A2 (5–20 μm) transcripts. Transcripts are plotted as black dots representing the covered genome positions and the % of identity with the closest reference. A horizontal grey line set at 95% identity shows the threshold used to count the number of times, or coverage, that a gene was expressed. The most expressed genes in both lineages are highlighted. (**b**) Relative contribution of nitrogen fixation operon, FOF1-ATP synthase operon, cytochrome b_6_f and PSI genes to the total UCYN-A transcripts contribution in surface samples; percentages are indicated. (**c**) Transcript counts of nitrogen fixation operon versus those of ATP synthase (triangle), cytochrome b_6_f (square) and PSI (open circle) transcripts. All of these transcripts were significantly correlated (*P*<10^−5^) and regression lines, regression equations and *R*^2^ values are indicated in the figure.

The analyses of seven size-fractionated metatranscriptomes from two stations (TARA_078 and TARA_076) and depths (surface and DCM) allowed for the first time a whole-genome transcription profiling of these widely distributed diazotrophic cyanobacteria (Table 1). In surface waters, UCYN-A1 transcripts were in general more abundant than those from UCYN-A2, except in the 5–20 μm size fraction (TARA_078) in which the latter were dominant (Table 1). The gene expression of 1,131 and 1,179 protein-coding genes in UCYN-A1 (Supplementary Data 1) and UCYN-A2 (Supplementary Data 2), respectively, were examined. In both lineages, the nitrogen fixation operon, including the *nifH* gene, was the most highly expressed gene-cluster accounting for a quarter of the total transcripts (Fig. 2a,b). In the >0.8 μm size fraction (TARA_078), despite UCYN-A1 being more abundant than UCYN-A2, the expressed *nifH* transcripts per cell were almost two times higher for UCYN-A2 (648.33) than for UCYN-A1 (396.60; Supplementary Data 1 and 2). It is well known that biological nitrogen fixation has a high energetic cost (16 mol of ATP to generate 2 mol of ammonia). Notably, the F0F1-ATP synthase operon and genes encoding for the cytochrome b_6_f complex and photosystem I complex (PSI) were highly transcribed and positively correlated (*P*<10^−5^, *N*=6, linear regression analysis) with the nitrogen fixation operon transcript abundances (Fig. 2c). These findings suggest that the generation of reducing power and the ATP synthesis could be coupled to fuel the nitrogen fixation process in UCYN-A. Likewise, UCYN-A2 might have higher nitrogen fixation rates per cell than UCYN-A1 based on the higher number of *nifH* transcripts per cell. It is reasonable to assume that the differences in *nifH* gene expression between the UCYN-A lineages could simply reflect the differences in the cell size of their partners with differential nutrient requirements for growth. In addition, it has been indirectly demonstrated that the nitrogen fixation of UCYN-A supports the CO_2_ fixation of its prymnesiophyte partner^18^. Therefore, we hypothesize that the larger *B. bigelowii* host cell would meet its larger N nutrient requirements by partnering with a larger number of UCYN-A2 symbiotic cells.

Nitrogen-fixing microorganisms, and particularly cyanobacteria, should protect their nitrogenase from inactivation by oxygen. The absence of the ability to use photosystem II that evolves O_2_ explains why UCYN-A appears to fix N_2_ and express the nitrogenase genes during the day^19^. However, its association with an oxygen-evolving partner could make the nitrogenase enzyme in UCYN-A not completely safe from oxygen. We observed that the *sufB* gene (cysteine desulferase), involved in the assembly or repair of oxygen-labile iron–sulfur clusters under oxidative stress, was highly transcribed (Supplementary Data 1 and 2). It may be that UCYN-A requires high expression level of *sufB* genes to repair the nitrogenase enzyme from oxygenic inactivation, suggesting then a similar role than for the peroxidase genes found in their genomes^11^,^12^. Our findings reveal that UCYN-A lineages dedicate a large transcriptional investment to fix nitrogen representing the first whole-genome expression profiling in environmental UCYN-A populations.

### UCYN-A diverged during the late Cretaceous

Our findings on partner fidelity in UCYN-A point to the hypothesis of symbiont–host co-evolution^14^. To analyse the selection pressure and evolution of the protein-coding genes, we calculated the number of synonymous or silent (Ks) and non-synonymous (Ka, inducing amino-acid change) nucleotide substitutions^20^,^21^ for 887 protein-coding genes shared by the UCYN-A1 and UCYN-A2 genomes (Supplementary Data 3). The Ka/Ks ratio may offer important clues about the selection pressure where ratios <1 indicate purifying selection and ratios >1 point to positive selection^22^. We found that 873 out of the 887 protein-coding genes were under purifying selection (*P*<0.05, codon-based *Z*-test) (Supplementary Data 3). The 14 remaining genes also presented Ka/Ks<1 but were not statistically well supported (*P*>0.05). Purifying selection means that synonymous mutations are maintained, while non-synonymous mutations are continuously removed from the population. We did not detect signs of large-scale positive selection, that is, no apparent strong adaptation to novel niches in UCYN-A lineages, suggesting that the evolutionary forces for niche adaptation would act on the prymnesiophyte partners rather than on UCYN-A. Our results are consistent with the fact that UCYN-A2 lacks the same major pathways and proteins that are absent in UCYN-A1 (ref. 12), indicating then that the symbionts were genetically adapted to their hosts before they were separated by speciation.

**Figure 3:**
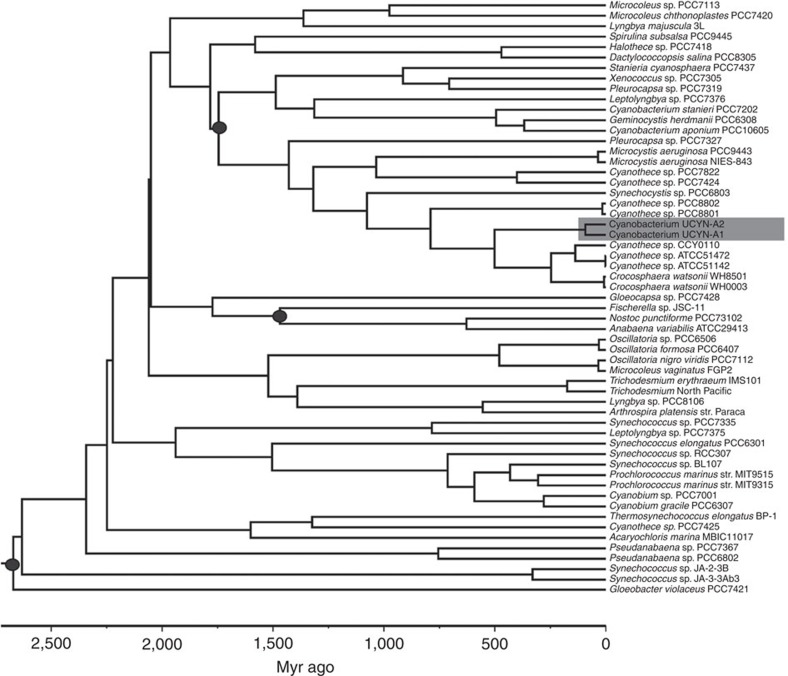
Time-calibrated cyanobacteria tree. The phylogeny shown was estimated based on 135 proteins from 57 taxa. Three calibration points (black circles) were used for the tree presented and were treated as soft bounds. The root of the tree was set with a maximum age of 2,700 Myr ago and a minimum age of 2,320 Myr ago. Divergence time for the ancestor of cyanobacteria UCYN-A1 and UCYN-A2 (highlighted with a grey box) is given with the corresponding values for the posterior 95% confidence intervals in Supplementary Table 2.

The age of divergence for UCYN-A1 and UCYN-A2 lineages was calculated by phylogenomic and Bayesian relaxed molecular clock analyses (Fig. 3; Supplementary Table 2). Our results indicate that UCYN-A1 and UCYN-A2 lineages diverged around 91 Myr ago, that is, during the late Cretaceous. In agreement, *B. bigelowii* has a fossil record extending back to the late Cretaceous (ca. 100 Myr ago)^23^, reported from neritic and pelagic sediments, for example, in lower Paleogene sediments immediately above the K/Pg mass extinction level as well as in the Oligocene Diversity Minimum^24^,^25^. In the Jurassic, between 190 and 100 Myr ago, nutrient availability in the ocean was lower than at any point during the last 550 Myr ago^26^. It is therefore likely that the symbiotic relationship between the common ancestor of UCYN-A1 and UCYN-A2, and a *Braarudosphaera*-related species was established by the late Cretaceous to cope with extremely low-nutrient conditions and a generalized oligotrophy in marine surface waters, as it has been recognized for other symbiotic system such as the Acantharia–*Phaeocystis* symbiosis^27^. UCYN-A then underwent purifying selection, progressively reducing its genome to the point that it became an obligate symbiont. An analogous discovery was the case of the two Rhopalodiaceae freshwater diatom species, *Rhopalodia gibba* and *Epithemia turgida* having acquired N_2_-fixing endosymbionts^28^,^29^. Similar to the two UCYN-A partnerships described here, phylogenies of these two diatoms species and their intracellular symbionts were found to be congruent and, concordantly, a single symbiotic event has been proposed^29^. Probably, a similar scenario can be envisioned here for the two UCYN-A partnerships.

Taking into account that the number of symbiotic cells harboured by distinct prymnesiophyte partners is different and phylogenetically dependent, that is, the larger *B. bigelowii* can harbour a variable number (up to 10) of UCYN-A2 cells, while the small prymnesiophyte partner harboured only one or two UCYN-A1 cells, it is reasonable to think that a larger nutrient acquisition could be linked to a larger number of symbionts. Indeed, the whole-genome expression patterns suggested that the metabolic investment in UCYN-A1 and UCYN-A2 is mainly focused on the nitrogen fixation machinery. Our evolutionary analysis revealed that UCYN-A1 and UCYN-A2 were genetically adapted to their prymnesiophyte partners before UCYN-A speciation (purifying selection) but, on the contrary, the prymnesiophyte partners seem to follow different ecological strategies^9^, suggesting a speciation process under positive selection. Our results suggest that the partner fidelity shown by UCYN-A lineages together with the speciation in the common ancestor of *B. bigelowii* and its closely related prymnesiophyte may have forced an allopatric speciation of UCYN-A1 and UCYN-A2 populations in the late Cretaceous. Comparative genome analysis of the two prymnesiophyte partners would clarify whether these two algal species underwent positive selection through evolution by adaptation to novel niches. As revealed by *nifH* phylogenetic analysis, it seems that novel UCYN-A lineages, such as UCYN-A3, and prymnesiophyte (or not prymnesiophyte) partners, will help to understand the evolutionary relationships of N_2_-fixing cyanobacterial symbionts and the extent of their ecological relevance on marine biogeochemical cycles.

The present study offers new insights into the marine nitrogen-fixing UCYN-A symbiosis by disentangling the partner fidelity, host–symbiont organization and size distribution, gene expression and evolution of UCYN-A1 and UCYN-A2 lineages. These results demonstrate that specific UCYN-A symbiotic pairs co-exist without cross-symbiotic partnerships. The fact that its distribution occupies new plankton size fractions accordantly to the host size should be considered in global nitrogen fixation models. The number of UCYN-A1 and UCYN-A2 cells involved in this symbiosis differs and appears to be a conserved phylogenetic trait. Remarkably, about a quarter of the UCYN-A transcripts were from nitrogen fixation genes, highlighting the importance of nitrogen fixation in this symbiosis. Our results present further evidences of a host and symbiont co-evolution scenario in the marine environment, probably derived from a single ancestral symbiotic event wherein at least two different lineages diversified in the late Cretaceous. Investigation of N_2_-fixing cyanobacterial symbionts and their partners should provide clues for discovering new ecological compartments for nitrogen fixation that would increase our understanding of the nitrogen cycle in the ocean.

## Methods

### Sample choice

From a total of 243 metagenomes from 68 globally distributed stations from *Tara* Oceans expedition^30^, the abundance of UCYN-A based on 16S _mi_TAGs^31^,^32^ and their corresponding prymnesiophyte partners evaluated by V9 18S iTAGS^9^,^33^, pointed out to a couple of stations, that is, TARA_078 (30° 8′ 12.12′′ S, 43° 17′ 23.64′′ W) and TARA_076 (20° 56′ 7.44′′ S, 35° 10′ 49.08′′ W) in the South Atlantic Ocean in which this symbiotic system were significantly abundant^9^, and therefore these two stations were chosen to further explore the UCYN-A symbiotic system.

### Sample collection

For the whole-cell CARD-FISH, 10 ml of surface seawater (pre-filtered with 20-μm pore-size mesh) was fixed with paraformaldehyde (1.5% final concentration) at 4 °C overnight and gently filtered through 0.2-μm pore-size polycarbonate filters (Millipore, GTTP, 25 mm diameter). For nucleic acid extractions and sequencing, surface seawater was collected and subsequently separated into four size fractions (0.2–3, 0.8–5, 5–20 and >0.8-μm pore-size filters)^34^,^32^. After filtration, filters were kept for ∼4 weeks at −20 °C on the schooner and then at −80 °C in the laboratory until processed for hybridization or nucleic acid extraction.

### Design of CARD-FISH probes

For the design of specific oligonucleotide probes targeting *B. bigelowii* and the closely related prymnesiophyte partner, a total of 580 sequences, 18S rRNA gene sequences, belonging to the class Prymnesiophyceae were retrieved from the PR2 database^35^, aligned using MAFFT^36^ and the alignment was verified manually to remove chimeras and sequences with ambiguities (466 sequences were kept). A maximum likelihood phylogenetic tree was built using RAxML^37^ with 100 trees for both topology and bootstrap analyses, and visualized with iTol^38^,^39^ (Supplementary Fig. 1). The newly designed probe UBRADO69 targeted *B. bigelowii*, while probe UPRYM69 targeted the closely related prymnesiophyte partner (Supplementary Table 1). UBRADO69 and UPRYM69 probes differed in only one position, and required a competitor to avoid unspecific hybridizations. Therefore, the labelled probe UBRADO69 was used in combination with the unlabelled UPRYM69 oligonucleotide for the detection of *B. bigelowii*, and vice versa for the detection of the closely related prymnesiophyte partner (Supplementary Table 1). Two helpers, helper-A PRYM and helper-B PRYM, were designed to improve the hybridization process for both probes (Supplementary Table 1). The UCYN-A732 probe designed against UCYN-A by targeting the 16S rRNA^15^ has only one mismatch with the UCYN-A2 sequence and a competitor was designed to distinguish specifically UCYN-A1 and UCYN-A2 clades with high specificity (Supplementary Table 1). The specificity of the new probes was checked with the online tool ProbeCheck (http://www.cme.msu.edu/RDP/) and by searching in the GenBank database (http://www.ncbi.nlm.nih.gov/index.html) to detect potential matching sequences in non-target groups.

### CARD-FISH assay and epifluorescence microscopy

A preliminary double-hybridization assay using the universal haptophyte PRYM02 probe^40^ and UCYN-A732 was first applied to check whether the partner of UCYN-A in our sample belong to class Prymnesiophyceae. To specifically target the different UCYN-A lineages and their prymnesiophyte hosts, a double-CARD-FISH assay was performed for each partnership (according to the multi-colour CARD-FISH protocol^41^). For the first hybridization step, the specific probe for one of the prymnesiophyte partners (UBRADO69 or UPRYM69) was used and, for the second step, the UCYN-A732 probe was used. To check the specificity of symbiont pairs, an additional double-CARD-FISH assay was carried out with the UBRADO69 probe and the UCYN-A732 as described before with the addition of the UCYN-A732 competitor to the hybridization buffer (probe, helpers and competitor at 0.16 ng μl^–1^). Filters were embedded in low-gelling-point agarose 0.1% (w/v) to minimize cell loss, and cell walls were permeabilized with lysozyme (37 °C, 1 h) and acromopeptidase solutions (37 °C, 0.5 h). For the first CARD-FISH step (described in more detail in Cabello *et al*.^9^), filters were hybridized overnight at 46 °C in 40% formamide (FA) hybridization buffer containing a mixture of the HRP (horseradish peroxidase)-labelled probe, helpers and competitor oligonucleotides. Filters were then rinsed in washing buffer at 48 °C and tyramide signal amplification was performed for 40 min at room temperature in the dark in a buffer containing 4 μg ml^−1^ Alexa 488-labelled tyramide. Before the second hybridization, the HRP from the first probe was inactivated with 0.01 M HCl for 10 min at room temperature in the dark^41^. The second CARD-FISH used the probe UCYN-A732 and its corresponding helpers and was applied according to Krupke *et al*.^15^. UCYN-A cells were hybridized for 3 h at 35 °C in 50% FA hybridization buffer, rinsed in washing buffer for 15 min at 37 °C and tyramide signal amplification was done as before but using 1 μg ml^−1^ Alexa 594-labelled tyramide. Preparations were counterstained with 4′,6-diamidino-2-phenylindole at 5 μg ml^−1^, mounted in antifading reagent (77% glycerol, 15% VECTASHIELD and 8% 20 × PBS) and kept frozen until microscopic analysis. A no-probe control showed that there was no signal coming from endogenous peroxidases. Filters were observed by epifluorescence microscopy (Olympus BX61) at 1,000 × under ultraviolet (4′,6-diamidino-2-phenylindole signal of the nucleous), blue-light (green-labelled host cells with Alexa 488) or green-light (red-labelled symbionts with Alexa 594) excitations. Micrographs were taken using an Olympus DP72 camera (Olympus America Inc.) attached to the microscope.

Hybridization conditions for the UPRYM69 and UBRADO69 probes were optimized testing different FA concentrations in the hybridization buffer and varying the hybridization temperature. The UPRYM69 probe (together with the competitor oligonucleotide) was tested in NE Atlantic surface samples, where UCYN-A1 host cells were ∼86% of prymnesiophytes (∼550 cells per ml). Initially, we tried 20–30–40–50% FA in the buffer and the temperature of 35 °C for hybridization. At 20% FA, host cells carrying UCYN-A (*n*=89) displayed a faint fluorescent signal (90%) or were not labelled (10%), whereas above 40% FA, no hybridized cells were detected. Signal was improved using helper oligonucleotides and host cells displayed a bright homogeneous signal at all FA concentrations, but we observed cross-hybridization (observed as fluorescent dots all over the cells) in larger prymnesiophyte-like cells not associated to UCYN-A even at 50% FA. Thus, we tested 40 and 50% FA in a hybridization temperature of 46 °C. The 40% FA showed optimal signal intensity, labelling small prymnesiophytes cells (∼2.5 μm) always carrying UCYN-A and no cross-hybridization was observed. We applied these conditions to hybridize the surface sample TARA_078. In this sample, in addition to the labelled small host cells observed in the NE Atlantic, we observed larger host cells not labelled by the UPRYM69 probe. To verify that these cells were the UCYN-A2 host, we applied the UBRADO69 probe with the same conditions (as both probes differ in only 1 position) and we found the complementary result: the larger host was labelled but not the smaller one. With the optimized conditions (40% FA, 46 °C), the probes were labelling specifically the target host without cross-hybridization.

### Nucleic acid extractions and sequencing

Surface and DCM seawater samples collected by *Tara* Oceans' station 76 and 78 in the South Atlantic Ocean (TARA_076 and TARA_078) for metagenomic sequencing were size fractionated. For surface samples, metagenomes from two and four fractions were analysed in TARA_076 (0.2–3 and >0.8 μm) and TARA_078 (0.2–3, 0.8–5, 5–20 and >0.8 μm), respectively. For DCM samples, metagenomes from one fraction were analysed in TARA_076 (>0.8 μm) and TARA_078 (>0.8 μm). Seawater samples for metatranscriptomic sequencing used also several size fractions. For surface samples, metatranscriptomes from two and three fractions were analysed in TARA_076 (0.2–3 and >0.8 μm) and TARA_078 (0.2–3, 5–20 and >0.8 μm), respectively. For DCM samples, metatranscriptomes from one fraction were analysed in TARA_076 (>0.8 μm) and TARA_078 (>0.8 μm). DNA and RNA extraction protocols for the different size fractions and metagenome sequencing are described in refs 31, 32, 33.

### cDNA synthesis and sequencing

For 0.2–3 μm and >0.8 μm filters, bacterial rRNA depletion was carried out on 240–500 ng total RNA using Ribo-Zero Magnetic Kit for Bacteria (Epicentre, Madison, WI). The Ribo-Zero depletion protocol was modified to be adapted to low RNA input amounts^42^. Depleted RNA was used to synthetize complementary DNA (cDNA) with SMARTer Stranded RNA-Seq Kit (Clontech, Mountain View, CA)^42^. For 5–20-μm filter from TARA_078, cDNA was synthetized starting from 50 ng total RNA using SMARTer Ultra Low RNA Kit (Clontech) by oligodT priming, following the manufacturer protocol. Full-length double-stranded cDNA was fragmented to a 150–600-bp size range using the E210 Covaris instrument (Covaris Inc., USA). Then, fragments were end-repaired and 3′-adenylated, and ligated to Illumina adaptors using NEBNext Sample Reagent Set (New England Biolabs, Ipswich, MA). Fragments were PCR-amplified using Illumina adapter-specific primers and purified. All metatranscriptomic libraries were quantified by qPCR using the KAPA Library Quantification Kit for Illumina Libraries (KapaBiosystems, Wilmington, MA) and library profiles were assessed using the DNA High Sensitivity LabChip kit on an Agilent Bioanalyzer (Agilent Technologies, Santa Clara, CA). Libraries were sequenced on Illumina HiSeq2000 instrument (Illumina, San Diego,CA) using 100 base-length read chemistry in a paired-end mode. Sequencing depth for each sample is detailed in Table 1.

### Nucleotide data deposition

Nucleotides data used in this study have been deposited in the European Nucleotide Archive (ENA; www.ebi.ac.uk/ena) under the following accession numbers: ERR1001626-27, ERR1007415-18, ERR1013384-85, ERR599006, ERR599010, ERR599022, ERR599126, ERR599237, ERR599240, ERR599250, ERR599253, ERR599275 and ERR599311.

### Fragment recruitment analysis from *omics* data sets

BLAST+ v2.2.25 was used to recruit metagenomic and metatranscriptomic reads similar to the two UCYN-A genomes sequenced up to date^11^,^12^ using default parameter values, except for the following: -perc_identity 50, -evalue 0.0001. Metagenomic/metatranscriptomic reads belonging to 23S, 16S and 5S rRNA genes or Internal Transcribed Spacer (ITS) regions as well as those aligned along <90% of its length were excluded (Table 1). The genome recovery was calculated as the percentage of nucleotide positions within the reference genomes aligned with metagenomic or metatranscriptomics reads >95% identity, threshold used for representing members of the same population as the reference genome^43^ (Table 1). To assess the gene expression at the genome level, we first used the gene positions to count the number of metatranscripts covering each gene. Then, we normalized these counts using two approaches (i) by UCYN-A single-copy house-keeping genes (*recA* and *gyrB* metatranscript counts), and (ii) by metagenomic read counts for each UCYN-A gene (in this case, we also normalized by sequencing depth; Supplementary Tables 2 and 3).

### Phylogenomic and relaxed molecular clock analyses

Sequence data for 57 cyanobacterial genomes were used to estimate the phylogenetic relationships of UCYN-A1 (ref. 11) and UCYN-A2 (ref. 12). We analysed 135 protein sequences that have shown to be highly conserved, to have undergone a minimum number of gene duplications and also to represent a wide diversity of cellular functions^44^. Maximum likelihood analyses and bootstrap values were performed using RAxML 7.4.2 (ref. 37). Bayesian relaxed molecular clock analyses as implemented in MCMCTree^45^ and PhyloBayes 3.3b^46^ were performed to estimate divergence times of UCYN-A1 and UCYN-A2 (Supplementary Table 2). We applied the uncorrelated gamma multipliers model^47^, as this model seems to fit better cyanobacteria nucleotide data sets based on Bayes factors^48^. Age divergences for UCYN-A1 and UCYN-A2 were estimated based on three genes: LSU (3,002 characters), SSU (1,546 characters) and *rpoC1* (1,887 characters). In PhyloBayes^46^, we implemented the CAT-GT replacement model of nucleotide evolution. For all non-calibrated nodes, we used a birth–death prior^49^ on divergence times. A permissive gamma distributed root prior of 2,500 Myr ago was also implemented (s.d.=200 Myr ago, which allowed the 95% credibility interval of the root node to range between 2,300 and 2,700 Myr ago). We treated all calibrations as soft allowing for 2.5% on each side for an upper and lower bound. In MCMCTree, LSU, SSU and *rpoC1* were treated as separate loci and branch lengths were estimated in BASEML^45^. We used the HKY85 (ref. 50) model of nucleotide evolution based on Bayes factor analyses^48^. We used 1 billion years per unit time for all analyses. The gamma prior G (*α* and *β*) used to describe how variable rates are across branches was specified as follows G (1, 7). The mean and s.d. was specified as *m*=*α*/*β*. The gamma priors for the substitution model parameters *κ* (transition/transversion rate ratio) and *α* (gamma shape parameter for variable rates among sites) were all specified by gamma distributions. Respective means and s.d.'s were (6, 2) for *κ* and (1, 1) for *α*. For all analyses, we used fixed values for the birth–death process *λ*=*μ*=1 and *ρ*=0. Analyses were performed at least twice to ensure convergence of the MCMC, although only one analysis is reported. For all age calibrations, both minimum and maximum bounds were soft and specified by uniform distributions between the maximum/minimum time constraints with 2.5% tail probabilities above/below these limits allowing for molecular data to correct for conflicting fossil information^51^. To check whether analyses had converged, we used Tracer v1.5.0 (http://beast.bio.ed.ac.uk/Tracer). For the cyanobacterial root, 2,700 Myr ago^52^ and 2,320 Myr ago^53^ (the rise in atmospheric oxygen) were set as the maximum and minimum age, respectively. Other fossils exhibiting unique morphological features were assigned to well-supported groups such as the Nostocales^54^ and the clade containing two *Pleurocapsa* genomes (PCC 7319 and PCC 7327) in the Pleurocapsales^55^.

## Additional information

**How to cite this article:** Cornejo-Castillo, F. M. *et al*. Cyanobacterial symbionts diverged in the late Cretaceous towards lineage-specific nitrogen fixation factories in single-celled phytoplankton. *Nat. Commun.* 7:11071 doi: 10.1038/ncomms11071 (2016).

## Supplementary Material

Supplementary Information Supplementary Figure 1 and Supplementary Tables 1-2.

Supplementary Data 1 Detailed list of genes expressed in UCYN-A1.

Supplementary Data 2 Detailed list of genes expressed in UCYN-A2.

Supplementary Data 3 Comparative genome analyses of UCYN-A lineages.

## Figures and Tables

**Table 1:** Fragment recruitment (FR) of UCYN-A lineages.

Station	Depth	Sample	Fraction	Sequencing	FR (reads)		Genome recovery (%)	
			(μm)	depth (reads)	UCYN-A1	UCYN-A2	UCYN-A1	UCYN-A2
76	SRF	MG	0.2–3	177,019,968	188,088	26	99.30	0.14
76	SRF	MT	0.2–3	18,908,305	25,340	137	21.35	0.37
76	SRF	MG	>0.8	73,651,199	54,776	147	98.61	1.35
76	SRF	MT	>0.8	10,283,396	12,143	322	15.01	0.59
76	DCM	MG	>0.8	115,099,936	848	3	9.00	0.03
76	DCM	MT	>0.8	12,998,358	76	3	0.49	0.02
78	SRF	MG	0.2–3	155,580,203	842,234	2,395	99.94	13.95
78	SRF	MT	0.2–3	13,151,362	133,693	453	46.61	0.99
78	SRF	MG	0.8–5	105,731,269	980,895	24,021	99.81	90.24
78	SRF	MG	5–20	139,070,786	1,182	17,028	10.14	76.47
78	SRF	MT*	5–20	97,646,287	292	17,862	1.76	34.69
78	SRF	MG	>0.8	163,575,710	719,803	81,528	99.32	99.03
78	SRF	MT	>0.8	9,966,043	44,613	9,415	30.77	11.51
78	DCM	MG	>0.8	86,446,300	1,358	45	13.32	0.48
78	DCM	MT	>0.8	10,659,304	82	10	0.71	0.07

DCM, deep chlorophyll maximum; MG, metagenome; MT, metatranscriptome; SRF, surface.

^*^A protocol that selectively sequenced RNA sequences with poly(A) tails was conducted.
