# Supplementary material for: Cyanobacterial symbionts diverged in the late Cretaceous towards lineage-specific nitrogen fixation factories in single-celled phytoplankton
Source: Nat Commun. 2016 Mar 22;7:11071. doi: 10.1038/ncomms11071 (PMC4804200; doi:10.1038/ncomms11071)
Supplement: Supplementary Information — Supplementary Figure 1 and Supplementary Tables 1-2. [file ncomms11071-s1.pdf]

Supplementary Fig 1.

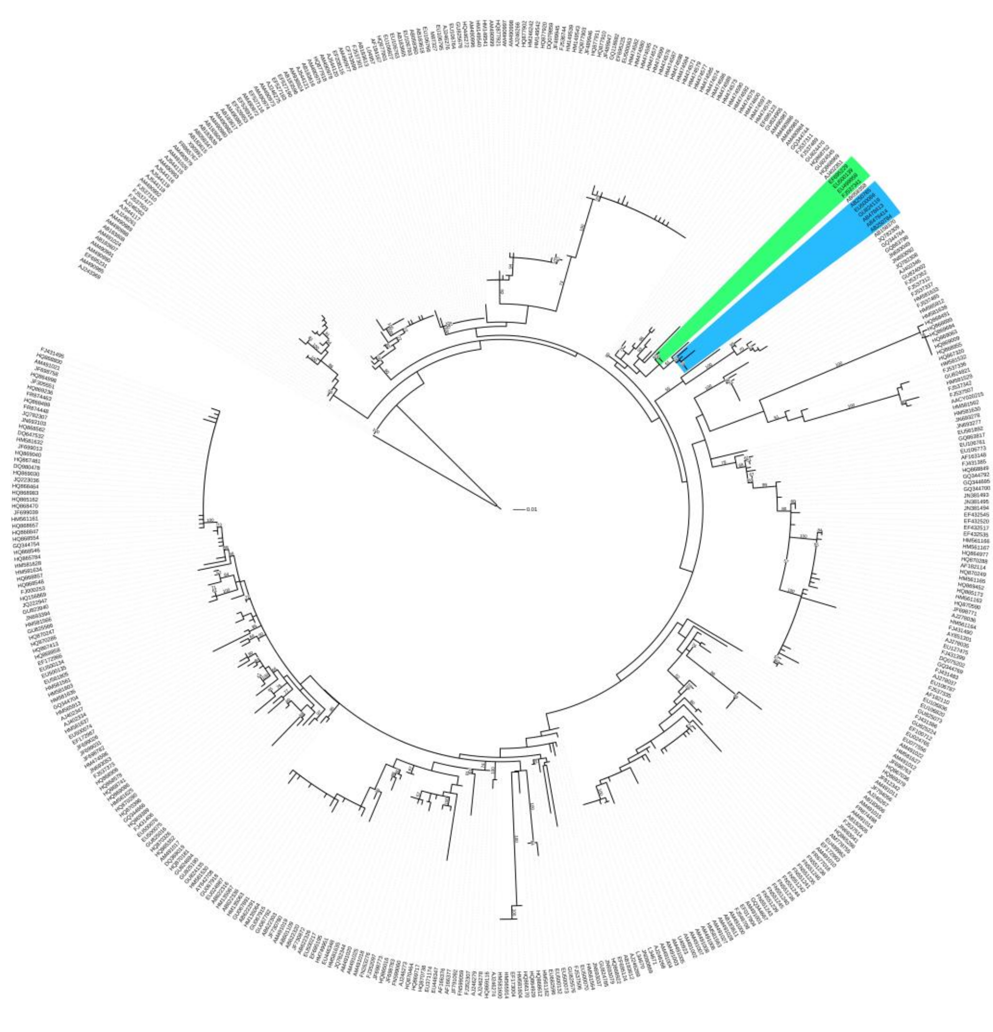

Supplementary Fig 1. Phylogenetic reconstruction of Class Prymnesiophyceae. Maximum likelihood phylogenetic tree of the Class Prymnesiophyceae based on the 18S rRNA gene. The tree includes 466 sequences (shown by their NCBI accession numbers) retrieved from the Protist Ribosomal Reference database (PR^2). Bootstrap values above 50% are indicated. The UCYN-A1 host phylogroup targeted by probe UPRYM69 probe is highlighted in green while the UCYN-A2 host phylogroup targeted by probe UBRADO69 is highlighted in blue.

**Supplementary Table 1 | Oligonucleotide probes used in CARD-FISH assays**			
**Probe**	**Target organism**	**Sequence (5' to 3')**	**Reference**
UCYN–A732	Unicellular cyanobacteria UCYN-A1	GTTACGGTCCAGTAGCAC	Krupke et al. (2013)
UCYN–A732 competitor	Unicellular cyanobacteria UCYN-A2	GTTGCGGTCCAGTAGCAC	This study
Helper A–732	Unicellular cyanobacteria UCYN-A	GCCTTCGCCACCGATGTTCTT	Krupke et al. (2013)
Helper B–732	Unicellular cyanobacteria UCYN-A	AGCTTTCGTCCCTGAGTGTCA	Krupke et al. (2013)
PRYM02	Prymnesiophyceae	GGAATACGAGTGCCCCTGAC	Simon et al. (2000)
UPRYM69*	UCYN-A1 host	CACATAGGAACATCCTCC	This study
UBRADO69*	*B. bigelowii*	CACATTGGAACATCCTCC	This study
Helper A-PRYM	Prymnesiophyceae	GAAAGGTGCTGAAGGAGT	This study
Helper B-PRYM	Prymnesiophyceae	AATCCCTAGTCGGCATGG	This study

*also used as competitor.

**Supplementary Table 2 | Divergence time for the ancestor of cyanobacterium UCYN-A1 and UCYN-A2**		
	**Phylobayes**	**MCMCtree**
**Divergence**	Independent	Independent
**UCYNA-1 and -2***	91 (46, 345)	141 (75, 234)

*Values in parenthesis correspond to the posterior 95% confidence intervals associated with median age estimates. Posterior age estimate in Million years.
